# The relationship of small vessel disease burden on cerebral and regional brain atrophy rates and cognitive performance over one year of follow-up after transient ischemic attack

**DOI:** 10.3389/fneur.2023.1277765

**Published:** 2023-11-24

**Authors:** Noaah Reaume, Meaghan Reid, George S. Tadros, Dorothy Chacinski, Britney Denroche, Arooj Aftab, Pauline Wu, Rani Gupta Sah, Meng Wang, Eric E. Smith, Richard Frayne, Shelagh B. Coutts, Tolulope Sajobi, Stewart Longman, Aravind Ganesh, Philip A. Barber

**Affiliations:** ^1^Calgary Stroke Program, Department of Clinical Neurosciences, Foothills Medical Centre, Calgary, AB, Canada; ^2^Seaman Family MR Center, Foothills Medical Centre, Calgary, AB, Canada; ^3^Cumming School of Medicine, Hotchkiss Brain Institute, University of Calgary, Calgary, AB, Canada; ^4^Department of Psychology, University of Calgary, Calgary, AB, Canada; ^5^Department of Medical Sciences, University of Calgary, Calgary, AB, Canada; ^6^Department of Radiology, University of Calgary, Calgary, AB, Canada; ^7^Department of Neurosciences, University of Calgary, Calgary, AB, Canada; ^8^Department of Community Health Sciences, University of Calgary, Calgary, AB, Canada

**Keywords:** brain atrophy, transient ischaemic attack, cerebral small vessel disease, cognitive profiles, magnetic resonance imaging

## Abstract

**Background:**

Stroke, even when minor, increases the risk of dementia. We aimed to determine whether patients with transient ischaemic attack (TIA) exhibit higher rates of cerebral and regional atrophy 1-year after first stroke symptoms and evaluate the relationship with small vessel disease and cognitive performance.

**Methods:**

TIA patients and controls without cognitive symptoms underwent high-resolution T1-weighted MRI and cognitive testing at baseline and 1-year. Percent brain volume change (PBVC) was measured, and the location of regional atrophy and small vessel disease (CSVD) burden was evaluated. Neuropsychological testing assessed memory, processing speed, and executive function.

**Results:**

A total of 76 TIA patients and 53 controls of mean age 67 (SD = 8) and 68 years (SD = 8) were recruited. TIA patients demonstrated greater improvement of visual memory and executive function at 1-year. TIA patients had greater median PBVC/year compared to controls (−0.79% [(−1.22)-(−0.38)] vs. -0.41% [(−0.62)-0.19]; *p* < 0.001), and higher rates of volume loss (ml/year) in subcortical gray (−0.53 [(−1.09)-(−0.06)] vs. -0.13 [(−0.61)-0.31]; *p* < 0.05) and white matter (−2.21 [−5.47, 0.40] vs. -0.93 [(−3.43)-2.10]; *p* < 0.05). Linear regression showed that TIA, age, and systolic blood pressure (SBP) were associated with greater cerebral volume loss over 1-year. There was no significant relationship between PBVC and 1-year cognition.

**Conclusion:**

A near two-fold increase in rate of cerebral atrophy 1-year after TIA is associated with higher SBP emphasizing the need for improved treatment of SBP. Cerebral and regional atrophy rates may be used to select patients for vascular risk reduction trials or novel therapeutics in future dementia prevention trials.

## 1 Introduction

Mixed dementia is the most common form of dementia in the older population, with incidence increasing with age (1). It is now generally accepted that a significant contributor to the failure of therapeutic treatments for dementia is that diagnosis can only be reliably established late in the disease course, following years of neurodegeneration and small vessel disease (2). Stroke is a distinct and important risk factor for dementia, doubling the risk of later cognitive decline independent of known risk factors for dementia (3). Furthermore, some patients with transient ischaemic attack (TIA) who are presumed to have fully recovered and have had vascular risks assessed and managed, nonetheless have mild detectable cognitive deficits that may persist. Additionally, a diagnosis of TIA is associated with a five-fold increase in age and sex-adjusted dementia incidence 1-year post-event and 15–20% incidence of dementia after 5 years (4, 5). A plausible explanation is that stroke may trigger specific mechanisms initiating or accelerating neurodegeneration, with increased small vessel disease burden (6) and inflammation (7) proposed as possible contributors.

The identification of reliable neuroimaging markers for those at risk of developing dementia may allow for the implementation of therapeutic and pharmacological interventions in the earliest stages of the disease, prior to symptom presentation. One proposed framework supplements established neuroimaging markers of neurodegeneration with biomarkers of vascular disease (e.g., small vessel disease, cortical and sub-cortical stroke) (8). Increased macroscopic brain loss, as measured through serial magnetic resonance imaging (MRI), is one such established marker of neurodegeneration associated with cognitive decline in mild cognitive impairment (MCI) and Alzheimer’s disease (AD) (9–11). Microscopic lesions such as white matter hyperintensities (WMH), micro-bleeds, lacunes, and enlarged perivascular spaces have been linked to cognitive outcome in population-based studies (12).

In this longitudinal cohort study of cognitively normal TIA patients, we primarily aimed to determine the annualized rate of percent brain volume change (PBVC), prior ventricular volume change (PVVC) and regional gray matter (GM) and white matter (WM) changes, as measured by serial high-resolution T1-MRI, in TIA participants and age-matched controls, and assess the relationship of PBVC in this cohort with vascular risk factor control, small vessel disease burden, and cognitive performance over 1-year.

## 2 Materials and methods

### 2.1 Study population

Consecutive high-risk TIA participants presenting with their first documented episode of transient stroke symptoms that fully resolved (13) in the anterior circulation (aphasia, motor weakness) or posterior circulation (visual field deficit, ataxia/vertigo plus one or more of motor, diplopia, hemiataxia) and control volunteers between the ages of 50–80 years were prospectively recruited between April 2015 and February 2019 (14) following written informed consent. Patients were diagnosed as TIA by a stroke neurologist. Subsequently, patients were reviewed by the senior neurologist (PAB) to ensure the inclusion criteria were met. Control participants were recruited from the community (e.g., hospital and community advertisements) and spousal partners. Ethics approval was provided by the University of Calgary Conjoint Health Research Ethics Board (REB13-0240).

### 2.2 Clinical data collection

The study design included a medical evaluation (clinical characteristics, demographics, medical history, medications, blood pressure) and neuropsychological testing, described in detail elsewhere (14). Both TIA patients and controls underwent the same evaluation. Premorbid intellect was calculated using the North American Adult Reading Test (NAART) (14). A total of 153 consecutive participants were identified with baseline and follow-up MRI of adequate quality. The patients with TIA were age matched with control subjects by either a random 1:1 or 2:1 matching procedure using STATA 15.0. This resulted in a final 129 participants included in the analysis (Supplementary Figure S1). Excluded participants did not differ from included participants with respect to age or sex distribution (*p* = 0.467, *p* = 0.129); however, excluded participants had, on average, lower baseline systolic blood pressure (*p* = 0.014). Smoking risk was defined by current or previous regular use.

### 2.3 Image acquisition

All participants underwent MR imaging at baseline and follow-up using a 3.0 T MR scanner with a standard multi-channel head coil (General Electric Discovery 750). TIA patients were imaged within 10 days of their presentation. T1-weighted, T2-weighted fluid-attenuated recovery (FLAIR), and diffusion weighted imaging (DWI) sequences were acquired for this analysis, with the same imaging protocol used at both time points [specific acquisition parameters are described elsewhere (8)]. Quality assurance to detect image distortion was conducted to assess the adequacy of key image contrast and signal-to-noise.

### 2.4 Image processing

#### 2.4.1 Percent brain volume, ventricular volume change and regional volume analysis

Brain tissue volume, normalized for subject head size, was measured with the FSL tool SIENAX (15), [RRID:SCR_002823]. Percent brain volume change (PBVC) was measured with the FSL tool SIENA (16), [RRID:SCR_002823]. The percent ventricular volume change (PVVC) was calculated with the SIENA extension, VIENA (16), [RRID:SCR_002823], which calculates the average brain edge displacement that is converted to the PVVC using a volumetric scaling factor (outputted from SIENA) (17). PBVC and PVVC were annualized for each participant, based upon the number of days between scans, to account for variability in follow-up time. Quality control was conducted at each time point during analysis to ensure accurate brain extraction, registration, and segmentation. Regional anatomical volumes were measured using FreeSurfer (18, 19); [RRID:SCR_001847]. Specifically, the longitudinal FreeSurfer [RRID:SCR_001847] stream was used to obtain and calculate reliable intra-subject volumes for the regions of interest, namely cortical GM, subcortical WM, and subcortical GM (20).

#### 2.4.2 Measurement of small vessel disease burden

Cerebral small vessel disease (CSVD) burden, as recommended by current STRIVE-2 guidelines (21), including white matter hyperintensities, chronic subcortical infarcts, enlarged perivascular spaces, and microbleeds, was rated visually on an ordinal scale from 0 to 6 using a previously validated semi-quantitative scoring system (22, 23). Using this scoring system, two neurologists (PAB, AG) rated images for the presence of cerebral microbleeds (CMBs), lacunes, white matter hyperintensities (WMHs), and enlarged perivascular spaces (EPVS). Additionally, white matter hyperintensities (WMH) were segmented from FLAIR images using Cerebra-WML, a semi-automated software that measures the volume of white matter lesions using the global threshold and contrast between different cerebral anatomical regions (24).

#### 2.4.3 Diffusion lesions

Diffusion lesions were segmented by trained research assistants (MR, NR) from DWI images with ITK-SNAP [Version 3.6.0^1^, RRID:SCR_002010], a semi-automated software used to segment structures in 3D medical images (25). DWI lesions were defined as per the STRIVE-2 guidelines (21).

### 2.5 Statistical analysis

Sample characteristics were described using descriptive statistics and frequency distributions. For crude group demographic comparisons, cognitive tests, and MRI metrics, Wilcoxon Rank Sum and Chi-square tests were used for continuous and categorical variables, respectively. Wilcoxon Signed Rank tests were used to compare total CSVD burden between baseline and follow-up, irrespective of group. Spearman correlations were performed to assess the relationship between DWI lesion volume and annualized PBVC/PVVC, and regional volume changes, among DWI-positive TIA patients only. Bonferroni corrections were employed when necessary to adjust for multiple comparisons.

Multiple linear regression models were performed to examine the association between TIA status and annualized performance on cognitive tests, while controlling for baseline age, sex, normalized brain volume (NBV), premorbid intellect (NAART), systolic BP, HDL cholesterol, smoking history, DWI volume, WMH volume, CSVD burden score. Moreover, multiple linear regression models were performed to examine the association between TIA status, annualized PBVC and PVVC, and the following covariates: baseline age, systolic blood pressure, HDL cholesterol, DWI volume, WMH volume, CVSD burden score change. Multiple linear regression models were also performed to investigate the association between annualized PBVC and cognitive scores at 1-year, after adjusting for TIA status, age, and sex. The assumptions of multiple linear regression models were not violated. All analyses were performed using IBM SPSS 28 [RRID:SCR_002865].

## 3 Results

At total of 129 age-matched subjects had follow-up MRI of adequate quality at 1-year. The TIA and control groups did not significantly differ with respect to age, sex, baseline depression symptoms, baseline HDL cholesterol, history of diabetes, or smoking history (current/past) (Table 1). Missing complete cognitive test data (TMT-B) at baseline (1 TIA) and follow-up (3 TIA, 2 controls) (due to refusal, fatigue, etc.) was excluded on a pairwise basis from analyses.

**Table 1:** Sample characteristics for TIA patients and controls (α = 0.05).

Characteristics	TIA (*n* = 76)	Control (*n* = 53)	*p*-value
Age, *years*	68.0 (61.0–75.0)	66.0 (60.5–73.0)	0.284
Sex, *n women* (%)	32 (42.1)	28 (52.8)	0.230
Education, *years*	13.5 (12.0–16.0)	15.0 (12.3–16.0)	**0.021**
Premorbid intellect (NAART)	108.7 (100.9–116.9)	113.6 (106.5–117.1)	**0.042**
Baseline to follow-Up, *days*	452 (376–495)	475.0 (421–582)	**0.038**
Baseline depression, *raw CES-D*	8.0 (3.0–16.0)	8.5 (4.0–14.0)	0.750
Systolic blood Pressure, mmHg*	140.7 (18.0)	127.6 (14.1)	**< 0.001**
HDL cholesterol, *mmol/L*	1.3 (1.1–1.6)	1.5 (1.1–1.7)	0.322
Hypertension treatment, *n* (%)	45 (59.2)	16 (30.2)	**< 0.001**
Diabetes, *n* (%)	7 (9.2)	1 (1.9)	0.090
Smoking, *n* (%)	39 (51.3)	20 (37.7)	0.128

NB: the reported numbers were medians with first quartile and third quartile unless otherwise stated; *Two-sample *T*-test used for systolic blood pressure and is presented as mean (SD). Bold values indicates significance of the alpha value.

NAART, North American Adult Reading Test; CES-D, Center for Epidemiological Studies-Depression Scale.

The cognitive profiles of participants are summarized in Table 2. At baseline, TIA participants scored significantly lower on the BVMT Delayed (*p* = 0.005), WAIS-IV Coding (*p* < 0.001), and TMT-B tasks (*p* = 0.004), however at follow-up, there were no significant differences in scoring between groups (Table 2). TIA status significantly predicted the rate of improvement over 1-year on the BVMT Delayed (*p* = 0.007) and WAIS-IV DS Coding tasks (*p* = 0.029), as compared to controls, after controlling for baseline risk factors.

**Table 2:** Wilcoxon Rank-Sum tests comparing median (Q1-Q3) performance of TIA and control participants in neuropsychological testing at baseline and follow-up (α = 0.05).

	Baseline			Follow-up		
	TIA	Control	*p*-value	TIA	Control	*p*-value
MMSE*	29.0 (28.0–30.0)	30.0 (30.0–30.0)	**< 0.001**	N/A	N/A	N/A
MoCA	25.0 (23.0–27.0)	27.0 (25.0–28.0)	**0.004**	26.0 (24.5–28.0)	27.0 (25.0–29.0)	0.115
ACE-R total	91.0 (88.0–96.0)	95.0 (91.0–98.0)	**< 0.001**	92.0 (88.0–95.0)	95.0 (91.0–98.0)	**0.009**
ACE-R verbal fluency	19.0 (15.0–21.0)	19.0 (19.0–21.0)	0.084	19.0 (15.0–21.0)	19.0 (15.0–21.0)	0.156
BVMT total	20.0 (15.0–25.0)	25.0 (19.0–30.0)	**< 0.001**	24.0 (20.0–29.0)	28.0 (23.0–31.0)	**0.020**
BVMT delayed	9.0 (7.0–11.0)	11.0 (9.0–12.0)	**0.005**	10.0 (8.0–11.5)	10.0 (9.0–12.0)	0.369
RAVLT	10.0 (7.0–12.0)	11.0 (7.0–13.0)	0.071	9.0 (7.0–12.0)	12.0 (9.0–14.0)	**0.008**
WAIS-IV DS coding	54.0 (48.0–63.0)	64.0 (57.0–73.5)	**< 0.001**	64.3 (53.0–78.0)	67.5 (57.0–79.0)	0.304
TMT-A**	32.1 (27.6–42.2)	32.0 (24.8–36.7)	0.431	32.7 (27.7–38.8)	29.6 (25.0–37.9)	0.194
TMT-B**	81.0 (64.5–99.6)	64.9 (53.2–85.1)	**0.004**	72.0 (57.3–102.0)	68.0 (53.4–81.8)	0.075

*MMSE only collected at baseline.

**TMT A and TMT B are reported as time (seconds) taken to complete the task.

Bold values indicates significance of the alpha value.

MMSE, Mini-Mental State Examination; MoCA, Montreal Cognitive Assessment; ACE-R, Addenbrooke’s Cognitive Examination-Revised; BVMT, Brief Visuospatial Memory Test; RAVLT, Rey Auditory Verbal Learning Test; WAIS-IV DS Coding, Wechsler Adult Intelligence Scale (4th Edition) Digit Symbol Coding; TMT, Trail Making Test.

The MRI metrics of participants are summarized in Table 3. There was a trend for greater baseline WMH volume in TIAs vs. controls (2.80 mL [1.78–5.60], 1.84 mL [1.15–4.44], *p* = 0.051). Participants did not differ with respect to annualized WMH volume change or CVSD burden score change over 1-year (*p* = 0.750, *p* = 0.756, respectively). However, total CSVD burden significantly increased between baseline (1.0 [0.0–2.0]) and follow-up (2.0 [1.0–3.0]), irrespective of group (*Z* = −5.904, *p* < 0.001).

**Table 3:** Baseline and follow-up MRI metrics of TIA and control participants compared using Wilcoxon-Rank Sum tests (α = 0.05).

	TIA (*n* = 76)		Control (*n* = 53)		
	Median	Q1-Q3	Median	Q1-Q3	*p*-value
NBV BL, *mL*	1441.19	1393.93–1486.27	1425.23	1356.18–1506.00	0.394
WMH BL, *mL*	2.80	1.78–5.60	1.84	1.15–4.44	0.051
WMH FU, *mL*	3.45	2.24–5.86	2.67	1.71–5.30	0.121
WMH change, *mL/year*	0.36	−0.06-0.98	0.30	−0.01-0.76	0.705
DWI volume BL, mL*	0.27	0.08–0.60	N/A	N/A	N/A
CSVD burden score change	0	0–1	0	0–1	0.977
PBVC, % *per year*	−0.79	−1.22-(−0.38)	−0.41	−0.62-(−0.19)	**< 0.001**
PVVC, % *per year*	4.99	2.21–7.57	3.75	0.64–6.10	0.064
Cortical GM change, % *per year*	−0.05	−0.44-0.28	0.17	−0.32-0.44	0.210
Cortical GM change, *mL/year*	−0.54	−5.69-3.36	3.18	−3.40-6.49	0.054
Subcortical GM change, % *per year*	−0.04	−0.09-(−0.01)	−0.01	−0.04-0.03	**0.011**
Subcortical GM change, *mL/year*	−0.53	−1.09-(0.06)	−0.13	−0.61-0.31	**0.011**
Subcortical WM change, % *per year*	−0.19	−0.43-0.04	−0.07	−0.29-0.15	**0.039**
Subcortical WM change, *mL/year*	−2.21	−5.47-0.40	−0.93	−3.43-2.10	**0.049**

*DWI volumes were only measured in 27 DWI-positive TIA patients. Bold values indicates significance of the alpha value.

BL, baseline; FU, follow-up; NBV, normalized brain volume; WMH, white matter hyperintensities; DWI, diffusion weighted image; CSVD, cerebral small vessel disease; PBVC, percent brain volume change; PVVC, percent ventricular volume change; GM, gray matter; WM, white matter.

TIA patients exhibited significantly higher annualized rates of percent brain volume change (PBVC/year), when compared to controls (−0.79%/year [−1.22-(−0.38)%/year], −0.41%/year [−0.62-(−0.19)%/year], respectively; *p* < 0.001) (Table 3). These values correspond to atrophy rates for TIA of −10.97 mL/year (−17.01-(−5.80) mL/year) and controls of −5.23 mL/year (−8.63-(−2.87) mL/year). There was a trend for greater PVVC in TIAs (+4.99%/year [2.21–7.57%/year], versus control values of +3.75%/year [0.64–6.10%/year]; *p* = 0.064).

There was no volumetric difference in segmented regions at baseline (cortical GM, subcortical GM, subcortical WM) between TIA and control participants (*p* = 0.109, *p* = 0.183, *p* = 0.639; corrected α=0.017 ). However, TIA participants were found to have higher rates of subcortical GM volume loss between baseline and follow-up, compared to controls (−0.53 mL/year [−1.09-(−0.06) mL/year], −0.13 mL/year [−0.61-(0.31) mL/year], respectively; *p* = 0.011). The TIA group also exhibited greater rates of volume loss in subcortical WM compared to controls (−2.21 mL/year [−5.47–0.40 mL/year], −0.93 mL/year, [(−3.43)–2.10 mL/year], *p* = 0.049).

Within the TIA group, 27 patients (35.5%) were found to be DWI-positive (0.27 mL, [0.08–0.60 mL]). The presence of a DWI-positive lesion was not significantly correlated with annualized PBVC or PVVC (*ρ* = −0.92, *p* = 0.650; *ρ* = 0.20, *p* = 0.320, respectively). DWI-positive TIA (*n* = 27) had an annualized PBVC of −0.95%/year (−1.23-(−0.32) %/year), while DWI-negative TIA (*n* = 49) had an annualized PBVC of −0.70%/year (−1.03-(−0.38) %/year). DWI lesion volume was not significantly correlated with regional volume changes (subcortical GM, *ρ* = −0.126, *p* = 0.530; subcortical WM, *ρ* = −0.323, *p* = 0.100).

Multiple linear regression results indicated an association between baseline risk factors and covariates and annualized rate of PBVC (Table 4). Within the model, TIA status (*p* = 0.040), greater age (*p* = 0.004) and baseline systolic blood pressure (*p* = 0.033) were significantly associated with increased annual PBVC, indicating a greater rate of whole brain volume loss. Finally, annualized PBVC was not found to significantly predict 1-year scores on any cognitive domain after adjusting for TIA status, age, and sex (*p* > 0.008 for all domains, adjusted for multiple comparisons, Table 5).

**Table 4:** Multiple linear regression model examining the associations between baseline risk factors (age, TIA status, systolic BP, HDL cholesterol, DWI volume, WMH volume, CSVD burden score change) and annualized rate of percent brain volume change (PBVC) (α = 0.05).

			Adjusted R-squared = 0.171 Model *p-*value <0.001
	Standardized β	95% CI	*p-*value
Age	−0.242	−0.360 – (−0.008)	**0.004**
TIA	−0.184	−0.408– (−0.076)	**0.040**
Systolic BP	−0.187	−0.396 – (−0.028)	**0.033**
HDL cholesterol	0.040	−0.347 – (−0.027)	0.626
DWI volume	−0.126	−0.293 – 0.040	0.136
WMH volume	−0.018	−0.198 – 0.153	0.827
CSVD burden score change	0.028	−0.135 – 0.191	0.733

NB: No potential collinearity among the independent variables was identified based on variance inflation factor and condition index. Bold values indicates significance of the alpha value.

**Table 5:** Multiple linear regression models examining the associations between annualized PBVC and cognitive scores within different cognitive domains at 1-year.

A. Visual memory			
	Standardized β	95% CI	*p-*value
BVMT delayed	0.130	−0.216-1.295	0.137

B. Verbal memory			
	Standardized β	95% CI	*p-*value
RAVLT	0.100	−0.402-1.478	0.202

C. Verbal fluency			
	Standardized β	95% CI	*p-*value
ACE-R verbal fluency	−0.027	−1.156-0.851	0.870

D. Processing speed			
	Standardized β	95% CI	*p-*value
TMT-A	0.025	−2.537-3.380	0.990

E. Executive function			
	Standardized β	95% CI	*p-*value
WAIS-IV DS coding	−0.076	−7.500-3.141	0.487
TMT-B	−0.067	13.067–5.961	0.370

Each cognitive score was tested in a separate model after adjusting for TIA status, age, and sex (α = 0.008, adjusted for multiple comparisons).

ACE-R, Addenbrooke’s Cognitive Examination-Revised; BVMT, Brief Visuospatial Memory Test; RAVLT, Rey Auditory Verbal Learning Test; WAIS-IV DS Coding, Wechsler Adult Intelligence Scale (4th Edition) Digit Symbol Coding; TMT, Trail Making Test.

## 4 Discussion

This study demonstrates that TIA participants experience near double the rate of annualized percent brain volume loss in the first year after the initial stroke event, compared to controls. Subsequent regional analyses suggest that this effect is related to increased rates of subcortical GM and WM volume losses over this period, in the absence of significant differences in cortical GM volume change between TIA and control groups. Increasing age, TIA status, and systolic blood pressure are associated with a greater rate of percent brain volume loss. DWI lesions, despite frequently being detected in TIA patients, were not associated with cerebral or regional rates of atrophy. The absence of a relationship between cerebral atrophy rates and DWI lesions supports our hypothesis that the observed brain volume changes are related to either chronic progressive incipient neurodegeneration, vascular mechanisms, or both (26).

Rates of whole-brain atrophy have been shown to parallel pre-clinical and clinical dementia progression, and is therefore frequently used as a disease biomarker for clinical trials (27). TIA participants had a median annualized PBVC of −0.79% *per annum* compared to −0.41% *per annum* for the control group. This is comparable to cerebral atrophy rates reported in mild cognitive impairment (MCI) (1.1% *per annum*) and in established AD (2–3% *per annum*) (10). In comparison, healthy controls experience an estimated cerebral atrophy rate of 0.2–0.5% *per annum* due to age-related decline, as is demonstrated by our control group (28). TIA patients therefore experience a lower atrophy rate than MCI patients, but a higher rate than healthy aging controls. It is, however, currently unknown if rigorous vascular risk reduction and lifestyle changes can limit this increased risk (4, 5). We found no significant relationship between cognition at 1-year and annualized PBVC, suggesting that structural changes can occur in the absence of short-term cognitive decline in TIA subjects (29). We found improvement on multiple cognitive tests in both TIA and control groups over 1 year follow-up. It is recognized that performance can improve after repeat cognitive testing even when alternate forms of the test are implemented (30). However, the TIA patients showed greater improvement than the controls on tests of visual spatial memory and attention, implicating not only practice effects, but also possible recovery mechanisms following the TIA.

Our results indicate that an increased rate of subcortical GM volume loss may be a significant component of the increased rate of whole-brain atrophy observed in our TIA cohort. Previous research has identified cross-sectional subcortical GM volume differences, in regions such as the hippocampus, amygdala, and nucleus accumbens, between MCI and controls (31). In patients with MCI, increased subcortical GM atrophy has been identified as a risk factor for AD conversion and has been shown to correlate with cognitive impairment severity (32).

Higher systolic blood pressure was found to be significantly associated with annualized PBVC in our cohort. Hypertension is a consistent risk factor for both stroke and dementia (30, 33). Hypertension has been associated with increased brain atrophy in cross-sectional (34) and longitudinal studies (29). There is a direct relationship between midlife hypertension, brain atrophy, and increased AD pathology (e.g., amyloid plaques, neurofibrillary tangles) at autopsy (35). The mechanisms by which hypertension affects cognitive function remain uncertain, but it is conceivable that high blood pressure may dysregulate cerebral blood flow resulting in regional hypoperfusion, ischemia, and inflammation (30, 33). This may lead to breakdown of the blood–brain barrier, contributing to progressive accumulation of small vessel disease and neurodegeneration (30, 33). Cerebrovascular disease has significant influence on cognitive performance in aging and commonly interacts with AD and other neurodegenerative diseases (36).

We did not find an association between WMH and cerebral atrophy rates, unlike previous studies of TIA and minor stroke (29), possibly because the relationship between WMH and cerebral atrophy is stronger when there is established stroke. Previous studies have found that increased WMH burden and decreased cerebrospinal fluid amyloid-beta levels are independently associated with greater brain atrophy in healthy control subjects, but not in MCI or AD patients, possibly because the latter two entities represent a more severe stage of disease (10). These results suggest that other sensitive markers of vascular disease, such as quantitative cerebral blood flow or white matter diffusion tensor imaging, should be measured to determine the relation of small vessel disease with brain atrophy rates (8). The analysis of total cerebral SVD burden demonstrated no significant differences between TIA and controls in our cohort at baseline, but further studies will be required to evaluate the relationship of cerebral SVD accumulation and how it contributes to measurable changes in brain volume over time. Previous studies have yielded mixed results on the relationship between markers of small vessel disease and volumetric cerebral atrophy changes (37). As there have been no previous comparisons of a global semi-quantitative CSVD burden score and rates of atrophy, there are opportunities for future subdomain analyses of this burden score (CMBs, lacunes, WMHs, EPVS) as they relate to brain atrophy.

This study has several potential limitations. We attempted to mitigate the impact of selection bias with a thorough description of the study cohort and the blinding of our analysis to group allocation. We also recognize the limitations of a relatively small sample size when utilizing regression analyses, with the risk of overfitting the statistical models. We also acknowledge that the inclusion of multiple outcome variables in our analyses may inflate the Type I error rate. The inclusion of healthy controls is limited by the challenges of identifying an appropriate match to participants in the disease group. However, the control outcome variables described in this study, including measures of brain atrophy, are in line with other published control data, indicating that these controls are similar to those included in other studies. Furthermore, we restricted our cohort to ages 50–80 resulting in a TIA cohort that is slightly younger, on average, than the general TIA population. This age range was chosen to evaluate younger patients presenting with early volumetric changes quantified by structural MRI and cognitive performance. We therefore acknowledge that these results may not be generalizable to the older patient.

This study has several important findings. First, we demonstrated a near two-fold increase in the rate of cerebral atrophy 1-year after TIA that is associated with higher systolic blood pressure, emphasizing the need for improved blood pressure control in high-risk populations. The increased rate of cerebral and regional brain atrophy occurred independently of the presence of a DWI lesion. Second, these changes in brain structure over 1-year occurred in the absence of significant cognitive change in association with PBVC, indicating that increased rates of brain atrophy occur prior to the development of clinically evident cognitive impairment. Therefore, and most importantly, this period of declining brain volume prior to cognitive change provides a window for preventative or novel therapeutics to mitigate the complex disease processes that may be contributing to the increased rate of brain atrophy. This study offers a unique insight into the early changes in brain volume detectable by MRI and the relationship with SBP even after a relatively short follow-up period of 1-year from the incident TIA. Predictors of early neurodegeneration, such as change in brain volume, are promising pre-clinical biomarkers that could monitor vascular risk management in the short-term, and ultimately improve subject selection strategies for future clinical trials.

## Data availability statement

The datasets presented in this study can be found in online repositories. The name of the repository and accession number can be found at: PRISM Data: University of Calgary’s Data Repository, https://borealisdata.ca/dataverse/calgary, DOI: 10.5683/SP3/DBJB1E.

## Ethics statement

The studies involving humans were approved by University of Calgary Conjoint Health Research Ethics Board (REB 13-0240). The studies were conducted in accordance with the local legislation and institutional requirements. The participants provided their written informed consent to participate in this study.

## Author contributions

NR: Data curation, Formal analysis, Project administration, Writing – original draft, Writing – review & editing. MR: Data curation, Formal analysis, Project administration, Writing – original draft, Writing – review & editing. GT: Data curation, Formal analysis, Writing – review & editing. DC: Data curation, Formal analysis, Writing – review & editing. BD: Data curation, Writing – review & editing. AA: Data curation, Writing – review & editing. PW: Data curation, Writing – review & editing. RG: Conceptualization, Project administration, Writing – review & editing. MW: Formal analysis, Writing – review & editing. ES: Conceptualization, Data curation, Project administration, Writing – review & editing. RF: Conceptualization, Formal analysis, Writing – review & editing. SC: Conceptualization, Writing – review & editing. TS: Conceptualization, Project administration, Writing – review & editing. SL: Data curation, Formal analysis, Methodology, Writing – review & editing. AG: Conceptualization, Data curation, Writing – review & editing. PB: Conceptualization, Data curation, Funding acquisition, Project administration, Writing – original draft, Writing – review & editing.

## Footnotes

^1^ www.itksnap.org

## Supplementary material

SUPPLEMENTARY FIGURE S1 Flow chart describing the age matching procedure and sample characteristics of included and excluded participants following matching.
