# Supplementary material for: The relationship of small vessel disease burden on cerebral and regional brain atrophy rates and cognitive performance over one year of follow-up after transient ischemic attack
Source: Front Neurol. 2023 Nov 24;14:1277765. doi: 10.3389/fneur.2023.1277765 (PMC10704595; doi:10.3389/fneur.2023.1277765)
Supplement: SUPPLEMENTARY FIGURE S1 — Flow chart describing the age matching procedure and sample characteristics of included and excluded participants following matching. [file Table_1.DOCX]

Supplementary Material

# Supplementary Figure

**
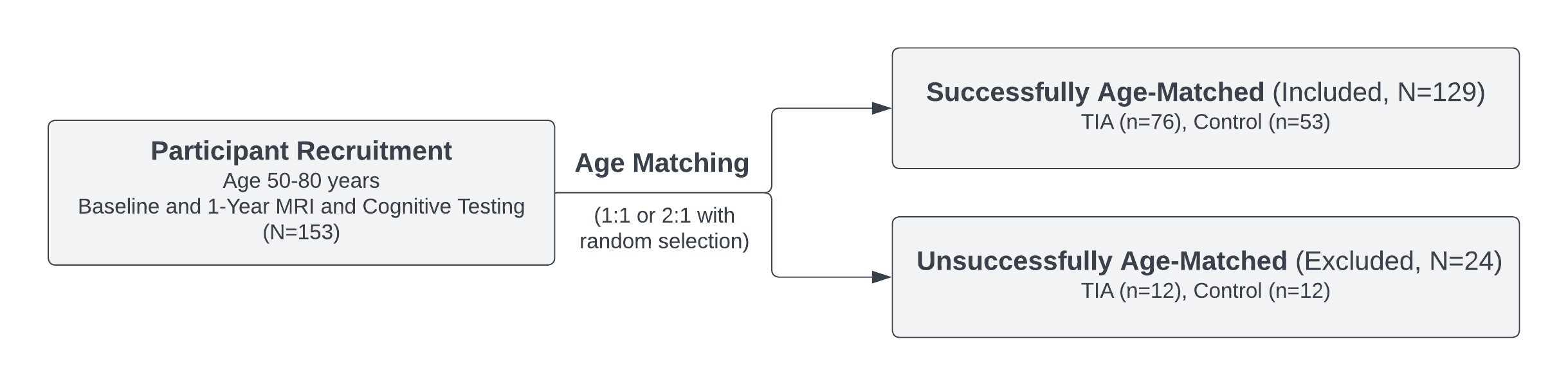
**

**Supplementary Figure 1.** Flow chart describing the age matching procedure and sample characteristics of included and excluded participants following matching.
